# Hippocampal Lesions can Enhance Discrimination Learning Despite Normal Sensitivity to Interference From Incidental Information

**DOI:** 10.1002/hipo.20995

**Published:** 2011-12-07

**Authors:** David J Sanderson, J Nicholas P Rawlins, Robert M J Deacon, Colm Cunningham, Chris Barkus, David M Bannerman

**Affiliations:** 1 Department of Experimental Psychology, University of OxfordOxford, United Kingdom; 2 School of Biochemistry and Immunology & Trinity College, Institute of Neuroscience, Trinity College DublinDublin, Republic of Ireland

**Keywords:** spatial learning, memory, mouse, lesion, conjunctive representations

## Abstract

Spatial properties of stimuli are sometimes encoded even when incidental to the demands of a particular learning task. Incidental encoding of spatial information may interfere with learning by (i) causing a failure to generalize learning between trials in which a cue is presented in different spatial locations and (ii) adding common spatial features to stimuli that predict different outcomes. Hippocampal lesions have been found to facilitate acquisition of certain tasks. This facilitation may occur because hippocampal lesions impair incidental encoding of spatial information that interferes with learning. To test this prediction mice with lesions of the hippocampus were trained on appetitive simple simultaneous discrimination tasks using inserts in the goal arms of a T-maze. It was found that hippocampal lesioned mice were facilitated at learning the discriminations, but they were sensitive to changes in spatial information in a manner that was similar to control mice. In a second experiment it was found that both control and hippocampal lesioned mice showed equivalent incidental encoding of egocentric spatial properties of the inserts, but both groups did not encode the allocentric information. These results demonstrate that mice show incidental encoding of egocentric spatial information that decreases the ability to solve simultaneous discrimination tasks. The normal egocentric spatial encoding in hippocampal lesioned mice contradicts theories of hippocampal function that suggest that the hippocampus is necessary for incidental learning per se, or is required for modulating stimulus representations based on the relevancy of information. The facilitated learning suggests that the hippocampal lesions can enhance learning of the same qualitative information as acquired by control mice. © 2011 Wiley Periodicals, Inc.

## INTRODUCTION

The spatial properties of stimuli are often encoded despite the spatial information being incidental to the demands of a particular learning task. For example, in a visual biconditional discrimination in which unique combinations of visual cues signal either reward or nonreward (e.g., AB+, CD+, AD−, CB−) rats are sensitive to the spatial structure of the cues such that if mirror images of the visual compounds are presented (e.g., BA+, DC+, DA−, BC−) then there is a reduction in the ability to solve the discrimination (Sanderson et al., 2006). This suggests that spatial information is automatically encoded (O'Keefe and Nadel, 1978; O'Reilly and Rudy, 2001).

While automatic encoding of spatial information may be useful for some forms of rapid new learning (Morris, 2006), it may interfere with acquisition of learning tasks in which a spatial solution is not necessary. Consider, for example, a simultaneous discrimination task in which a rewarded cue (A+) and a nonreward cue (B−) are presented in a spatial arrangement with each cue in a different location. Animals are required to make a choice by approaching or avoiding a particular cue. So that the choice of a cue is not confounded by choice of spatial location, the cues can be presented equally often in the two locations, thus making spatial stimuli irrelevant for the solution of the discrimination. However, if spatial properties of cues are automatically encoded then it is possible that any learning that occurs when a cue is in a particular spatial location (e.g., A+ on the left) will not fully generalize to when the cue is presented in the opposite location (e.g., A+ on the right). Also, the fact that the spatial information may be automatically encoded results in the addition of common cues, which will increase the difficulty of the discrimination. Thus, when stimuli that predict different outcomes are presented in the same location on different trials (e.g., A+ left, B− left) they share common spatial information that may increase their similarity.

Given the role of the hippocampus in spatial learning (O'Keefe and Nadel, 1978; Morris et al., 1982) it may be expected that lesions of the hippocampal system may facilitate certain learning tasks by reducing interference from spatial cues. Indeed, there are a number of reported facilitations following damage to the hippocampal system on tasks in which animals are required to simultaneously discriminate between cues that are presented in different locations (Meunier et al., 1996; Bussey et al., 1998; Saksida et al., 2007). Facilitations may occur for at least two reasons, (i) because hippocampal lesions impair encoding of the spatial properties of the predictive cues, or (ii) facilitated learning may also occur simply because normal animals persist with a spatial strategy before shifting to a cue-based strategy. Thus, impaired spatial learning may result in hippocampal lesioned animals adopting a cue-based strategy earlier in training. Any facilitation on simultaneous discrimination tasks that is caused by hippocampal lesions may be explained in terms of these two accounts. However, the two accounts lead to different predictions. If hippocampal damage facilitates discrimination learning by impairing encoding of the spatial properties of cues then the stimulus representations that are learnt during acquisition will be qualitatively different between hippocampal lesioned animals and normal animals. Alternatively, if hippocampal lesions result in animals adopting the correct cue-based strategy earlier on in training than in normal animals then at the end of training it would be expected that the facilitated learning would reflect a quantitative, rather than a qualitative difference in learning of the same stimulus representations.

**Figure 1:**
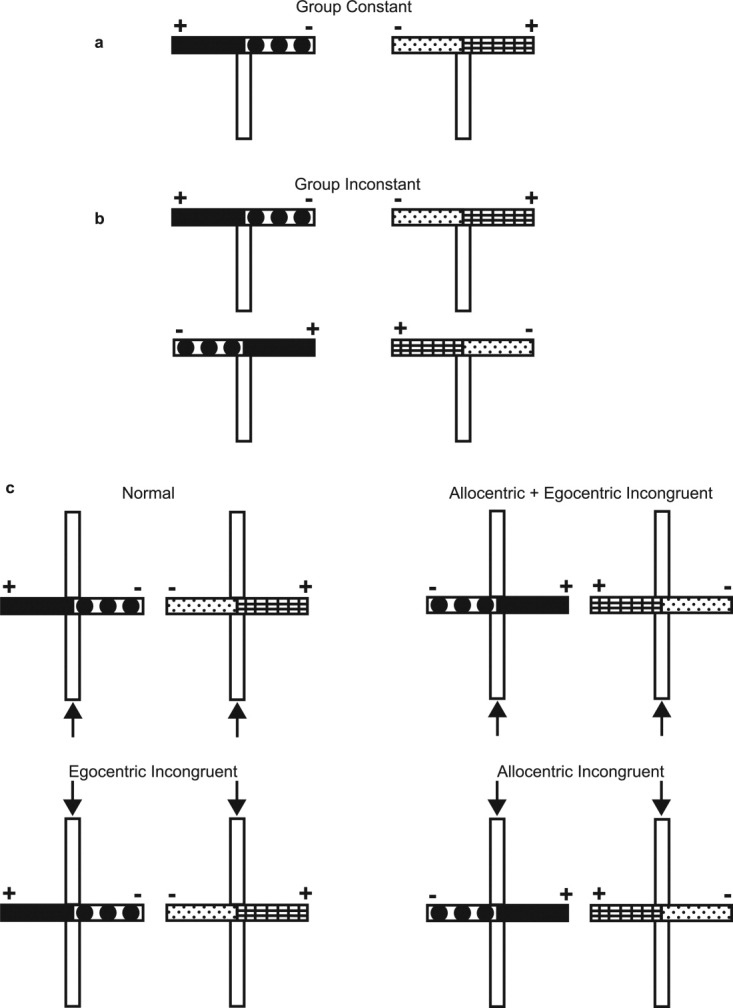
Design of Experiments 1 and 2. Pairs of inserts, made from different materials, were placed in the goal arms of a T-maze. For each pair of inserts, food reward was located at the end of one insert (indicated by the “+” sign) and no food was present at the end of the other insert (indicated by the “−” sign). (a) In Experiment 1, Group Constant received trials with two different pairs of inserts. For one insert pair the insert in the left goal arm was rewarded, but for the other pair the insert in the right goal arm was rewarded. (b) Group Inconstant received similar training to Group Constant, except that the rewarded insert for each insert pair was presented equally often in the left and in the right goal arm. At the end of the acquisition phase in Experiment 1 Group Constant (a) were switched to the same task as Group Inconstant (b, probe test 1). (c) In Experiment 2 mice were trained on the same task as Group Constant in Experiment 1 (a). At the end of acquisition mice received probe tests in which the spatial properties of the inserts were altered. This was done be either reversing the spatial location of the inserts in the left and right goal arms or by starting mice from the opposite start arm (i.e., either north or south), or both. For the Normal trials (c, top left) the inserts were placed in the same goal arms as used in acquisition and mice were started from the same start arm used in training (indicated by the arrows), thus keeping the allocentric and egocentric spatial information constant. For the Allocentric + Egocentric Incongruent trials (c, top right) mice were started from the same start arm as used in acquisition, but now the spatial locations of the inserts was reversed. This resulted in altering both the allocentric and egocentric spatial information. For the Egocentric Incongruent trials (c, bottom left) the inserts were placed in the same goal arms as used in acquisition, but now mice were started from the start arm that was opposite to that used in acquisition (indicated by the arrows). This resulted in keeping the allocentric spatial information constant, but altering the egocentric spatial information. For the Allocentric Incongruent trials (c, bottom right) the spatial locations of the inserts was reversed and mice started from the start arm that was opposite to that used in acquisition. This resulted in keeping the egocentric spatial information constant, but altering the allocentric spatial information.

In the following experiments the effects of hippocampal lesions in mice were tested on (i) acquisition of simultaneous discrimination learning and (ii) the incidental encoding of the spatial properties of cues. Mice were trained on simultaneous discrimination tasks using floor inserts in the goal arms of a T-maze. The design of Experiment 1 was based on experiments conducted by Bitterman and colleagues (Bitterman, 1952; Teas and Bitterman, 1952; Turbeville et al., 1952). Mice were divided into two groups (Groups Constant and Inconstant). Group Constant received training in which they presented with insert A in left goal arm (AL) and insert B in the right goal arm (BR) on half of the trials. On the remaining trials they were presented with insert C in the left goal arm (CL) and insert D in the right goal arm (DR, see Fig. 1a). Inserts A and D were rewarded and B and C were nonrewarded. This resulted in reward occurring in the left and right goal arms equally often over training (e.g., AL+ BR−, CL− DR+). Group Inconstant received similar training except that the insert pairs (A and B, C and D) were presented in the reverse spatial arrangement on half of the trials (e.g., AL+ BR−, BL− AR+, CL− DR+, DL+ CR−, see Fig. 1b). In both the Constant and Inconstant conditions spatial information is irrelevant for the solution of the task, but the two conditions differ in the degree that spatial information may interfere with learning. While interference that is caused by rewarded and nonrewarded inserts appearing in the same goal arms on different trials (e.g., AL+ CL−) should affect both groups equally, the groups will differ in whether they will be affected by generalization decrement caused by inserts appearing in different goal arms. In the Constant condition the inserts appear in only the left or right goal arm. In the Inconstant condition the inserts appear equally often in both the left and right goal arms. Generalization decrement that is caused by a cue appearing in different spatial locations will affect only the Inconstant condition, but not the Constant condition. This should result in slower learning in the Inconstant condition than in the Constant condition. If hippocampal lesions facilitate learning of simultaneous discriminations due to impairing encoding of spatial properties of cues then it would be expected that any facilitation would be greater in Group Inconstant than in Group Constant. Alternatively, if hippocampal lesioned mice adopt a cue-based strategy earlier in training then any facilitation would be equal in Group Constant and Inconstant.

In Experiment 1 two probe tests were carried out to assess the nature of learning in the different conditions. At the end of acquisition Group Constant were switched to the same task as Group Inconstant (probe test 1) so that they were now presented with the inserts in the opposite spatial arrangement (Fig. 1b). If performance was reduced when presented with these novel spatial arrangements this would provide direct evidence that mice had encoded the spatial properties of the stimuli. In probe test 2 both groups were presented with rewarded and nonrewarded cues in novel combinations (e.g., A+ and C−, D+ and B−) to assess whether mice had encoded the rewarded and nonrewarded cues as a configuration (Eichenbaum et al., 1989).

The purpose of Experiment 2 was to establish whether the incidental spatial information that was learnt reflected either allocentric or egocentric cues. Mice were trained on the same task as Group Constant in Experiment 1, but now using a cross-maze (Fig. 1a), which has been shown to permit sampling of extramaze, allocentric cues (Sanderson and Bannerman, 2011). At the end of acquisition mice received probe tests that assessed whether mice had encoded the allocentric or egocentric properties of the cues (see Fig. 1c).

## METHODS

### Subjects

Female C57BL/6J/Ola mice, obtained from Harlan OLAC, Oxon, UK were used in the following experiments. Mice were housed in group cages of 2–6 in a temperature controlled holding room on a controlled light-dark cycle (0700–1900) and had free access to food and water unless otherwise stated. Mice were ∼3 months old at the time of surgery and were ∼6 months old at the start of behavioral testing. Mice were maintained at 85% of their free-feeding weight during behavioral testing. Surgical procedures and stereotaxic coordinates for hippocampal lesioned mice (HPC) were the same as reported by Deacon and colleagues (Deacon et al., 2002). In Experiment 1 sham lesioned mice (Sham) underwent anesthesia (isoflurane, ∼2%) and received a craniotomy, but then received no more surgical procedures. In Experiment 2 sham lesioned mice underwent anesthesia and an incision was made to the scalp, but no craniotomy was performed. Mice were given perisurgical analgesia (carprophen 5 mg/kg), and chlordiazepoxide (CDZP; 10 mg/kg) and atropine (0.075 mg/kg) were given to minimize seizure activity and bronchial secretions, respectively. At the end of surgery mice were placed in a temperature (30°C) controlled recovery chamber until locomotor ability was regained. The mice were subsequently weighed daily until their weights had stabilized and given 10% glucose in the drinking water for 2–4 days postoperatively to aid recovery. At the end of behavioral testing the mice were anesthetized and perfused transcardially with physiological saline followed by 10% formol saline. The brains were removed and stored in formol saline. They were subsequently placed in 30% sucrose-formalin solution for 24 h, frozen, sectioned coronally (40 μm thick) and stained with cresyl violet.

Before discrimination training the mice used in Experiments 1 and 2 had been trained on spatial tasks to verify the efficacy of the lesions. Hippocampal lesioned mice used in Experiment 1 were significantly impaired on spatial alternation (see Sanderson et al., 2009) and the hippocampal lesioned mice used in Experiment 2 were significantly impaired on a spatial reference memory task (manuscript in preparation).

### Apparatus

Experiment 1 was conducted in a gray-painted, elevated, wooden T-maze that consisted of a start arm (47 × 10 cm) and two identical goal arms (35 × 10 cm) surrounded by a 10 cm high wall. Four wooden floor inserts (40 × 9.5 cm) covered with different materials acted as discriminanda signaling the location of the reward. The materials used to make the four distinct inserts were laminated black circles on a white background covered in wire mesh (W), green waterproof sandpaper with a white plastic border (1 cm wide; S), black rubber (R), and laminated black and white checked print (L). All inserts were symmetrical along their vertical and horizontal axes. When an insert was placed in each of the goal arms the two inserts met in the middle of the start arm at the choice point (see Fig. 1). The orientation of each insert within a goal arm changed in a pseudo-random order from session to session, so that each end of the insert was equally often located either at the end of the goal arm furthest from the start arm or at the choice point in the start arm. A metal food well (1 cm in diameter, 0.5 cm deep) was attached to the end of each insert located at the end of the goal arm. Correct choices were rewarded with 0.1 ml sweetened condensed milk (Nestle, York, U.K.) diluted 50% with water.

Experiment 2 was conducted in a cross-maze made from the same materials as the maze used in Experiment 1. The cross-maze had two start arms (North and South; 47 × 10 cm) and two goal arms (East and West; 35 × 10 cm). The entrance to one of the start arms could be blocked with a wooden block, thus creating a T-maze similar to that used in Experiment 1. Previous research has shown that C57BL/6J/Ola mice are able to sample extramaze, allocentric spatial cues when tested in this maze (Sanderson and Bannerman, 2011).

## EXPERIMENT 1

### Acquisition Phase

Mice were randomly assigned to either Group Constant (Sham, *N* = 12; HPC, *N* = 12) or Group Inconstant (Sham, *N* = 8; HPC, *N* = 10). Group Constant received discrimination training with two pairs of stimuli (1st pair, A+ vs. B−; 2nd pair, C− vs. D+, see Fig. 1a). For trials with the 1st pair, stimulus A+ was always located in the left goal arm and stimulus B− was always located in the right goal arm. For trials with the 2nd pair, stimulus C− was always located in the left goal arm and stimulus D+ was always located in the right goal arm. Stimuli A+ and D+ were rewarded, whereas B− and C− were not. Thus, each insert stimulus either predicts reward or nonreward, but the left and the right goal arms are equally rewarded and nonrewarded. Inserts W and S were used as one pair of stimuli and R and L were used as the other pair. For approximately half of each lesion group W and S were allocated as the 1st pair and R and L were allocated as the 2nd pair, and vice versa for the remaining mice. Within each of these subgroups, stimuli A+ and D+ (i.e., the rewarded inserts) were W and R, W and L, S and R, or S and L for approximately a quarter of the mice. Group Constant received 12 sessions, one per day. Each session consisted of four trials with each pair of stimuli (i.e., 1st and 2nd pairs), thus equalling eight trials, per session in total. The order of the trials was random with the constraint that there could be no more than three trials with the same pair of stimuli in consecutive order.

Group Inconstant received similar training to Group Constant except that stimuli A+ and B−, C− and D+ were placed equally often in the left and right goal arms (see Fig. 1b). Within each session Group Inconstant received two trials in which A+ was in the left goal arm and B− was in the right goal and two trials in which A+ and B− were in the opposite spatial arrangement. This was also true for C− and D+. Therefore, mice received eight trials per session in total. The order of the trials was random with the constraint that no more than three trials with the same pair of stimuli could occur in consecutive order. Also, there could be no more than three consecutive trials in which reward was presented in the same goal arm. All other details were the same as for Group Constant.

At the start of a trial the mouse was placed at the end of the start arm facing away from the goal arms. The mouse was allowed to traverse the start arm and enter one of the goal arms. Mice were considered to have made a choice once the end of their tail was beyond the entrance of a goal arm. If the mouse had chosen the correct stimulus it was allowed to consume the reward. If the mouse had chosen the incorrect stimulus it was immediately removed from the maze and returned to its home cage. The intertrial interval was approximately 5–10 min.

On the 10th session to assess whether the task was being solved by use of the inserts as opposed to smelling the food reward both the rewarded and nonrewarded inserts were baited with sweetened condensed milk. If mice made an incorrect choice they were immediately removed from the goal arm and were not allowed to drink the milk. During this session performance was maintained at a similar level to previous training and all mice performed above chance levels (*P* < 0.0005).

#### Probe test 1

In sessions 13–18 Group Constant now received discrimination training that was identical to Group Inconstant (see Fig. 1b). The performance of Group Constant on probe trials in which A+ and B−, and C− and D+ were now in the opposite arrangement was compared to performance of Group Inconstant on the equivalent trials determined by the counterbalancing of the insert allocations and reward contingencies.

#### Probe test 2

In sessions 19–20 both groups received probe trials with novel pairings of rewarded and nonrewarded inserts. Thus, stimuli from the 1st and 2nd insert pairs were intermixed (i.e., A+ and C−; B− and D+). Both groups received two trials of each of the original stimulus pairs (i.e., A+ and B−; and C− and D+) and two probe trials of each of the new stimulus pairs. On half of the trials with each stimulus pair the rewarded stimulus was presented in left goal arm, and in the right goal arm in the remaining trials.

## EXPERIMENT 2

### Acquisition Phase

Sham and hippocampal lesioned mice (*N* = 11 per group), different from those used in Experiment 1, received eight sessions of discrimination training in a similar manner to Group Constant in Experiment 1 (see Fig. 1c - Normal). Inserts W and S were used as the 1st stimulus pair and R and L as the 2nd stimulus pair. Within each lesion group, stimuli A+ and D+ (i.e., the rewarded inserts) were W and R, W and L, S and R, or S and L for approximately a quarter of the mice. Half of the mice allocated to each of the different insert pairings and reward contingencies were trained starting from the south start arm, and the remaining mice were trained starting from the north start arm. All other details were the same as for Experiment 1.

### Probe Test

In sessions 9–14 mice received three types of probe trials intermixed with normal trials (see Fig. 1c). For the first probe test trial type (Allocentric + Egocentric Incongruent) stimuli A+ and B−, and C− and D+ were presented in the opposite spatial arrangement to that used in acquisition (i.e., B− to the left of A+, D+ to the left of C−). Mice started from the same start arm used throughout acquisition (i.e., north or south start arm). Thus, these probe test trials were identical to probe test 1 in Experiment 1. For the second probe test trial type (Egocentric Incongruent) stimuli were presented in the normal spatial arrangement, as used in acquisition, but now mice started from the opposite start arm. Thus, although the cues remained in the same locations the relative egocentric spatial information was reversed. For the third probe test trial type (Allocentric Incongruent) stimuli were presented in the opposite spatial arrangement to that used in acquisition, but now mice also started from the opposite start arm to that used in acquisition. This resulted in maintaining the relative egocentric spatial information, but reversing the allocentric information.

Mice received one trial of each of the four trial types (i.e., Normal; Allocentric + Egocentric Incongruent; Egocentric Incongruent; Allocentric Incongruent) with each stimulus pair (i.e., eight trials in total) per session. Mice received six sessions of probe testing, thus totalling 12 trials of each trial type.

## RESULTS

### Histology

**Figure 2:**
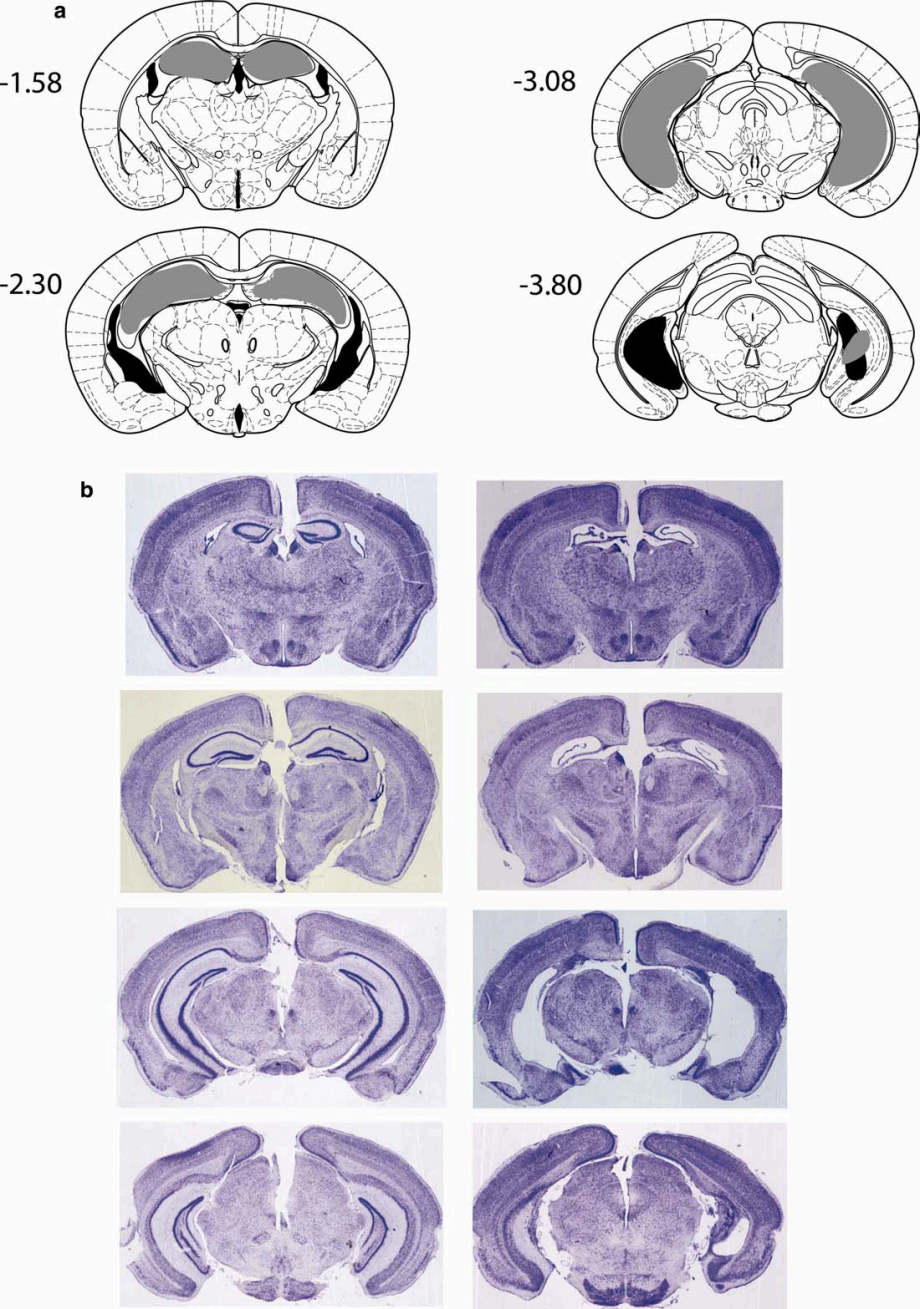
(a) Coronal sections (Franklin and Paxinos, 2001) of the hippocampus depicting the largest (black) and smallest (gray) hippocampal lesions. The numbers refer to the distance (in mm) posterior to bregma. (b) Photomicrographs of four coronal sections from a control brain (left) and a representative hippocampal lesion (right) in C57BL/6J/Ola mice. The sections (40 μm thick) correspond approximately to points (from top to bottom) −1.58, −2.30, −3.08, and −3.80 mm from bregma. [Color figure can be viewed in the online issue, which is available at wileyonlinelibrary.com.]

The histology for Experiment 1 has previously been reported (Sanderson et al., 2009). In brief, the hippocampus was almost entirely removed or remained only as damaged gliotic tissue in most cases. Three lesioned mice in Group Constant had unilateral sparing of the dorsal hippocampus and were consequently removed from all subsequent analyses (leaving *N* = 9). Small amounts of intact tissue remained in a minority of mice. This sparing was confined to the medial dentate gyrus and the posterior part of the ventral hippocampus. The hippocampal lesions in Experiment 2 were very similar to those in Experiment 1 (see Fig. 2). In most cases the lesions were very large, encompassing the whole of the dorsal hippocampus and with only small amounts of intact tissue in the extreme posterior part of the ventral hippocampus. Two mice had unilateral sparing of the dorsal hippocampus and were consequently removed from all subsequent analyses (leaving *N* = 9). In both experiments damage was restricted to the hippocampus, apart from small local damage around the needle tracts in the cortex overlying the hippocampus in a minority of mice.

### Experiment 1

#### Acquisition phase

**Figure 3:**
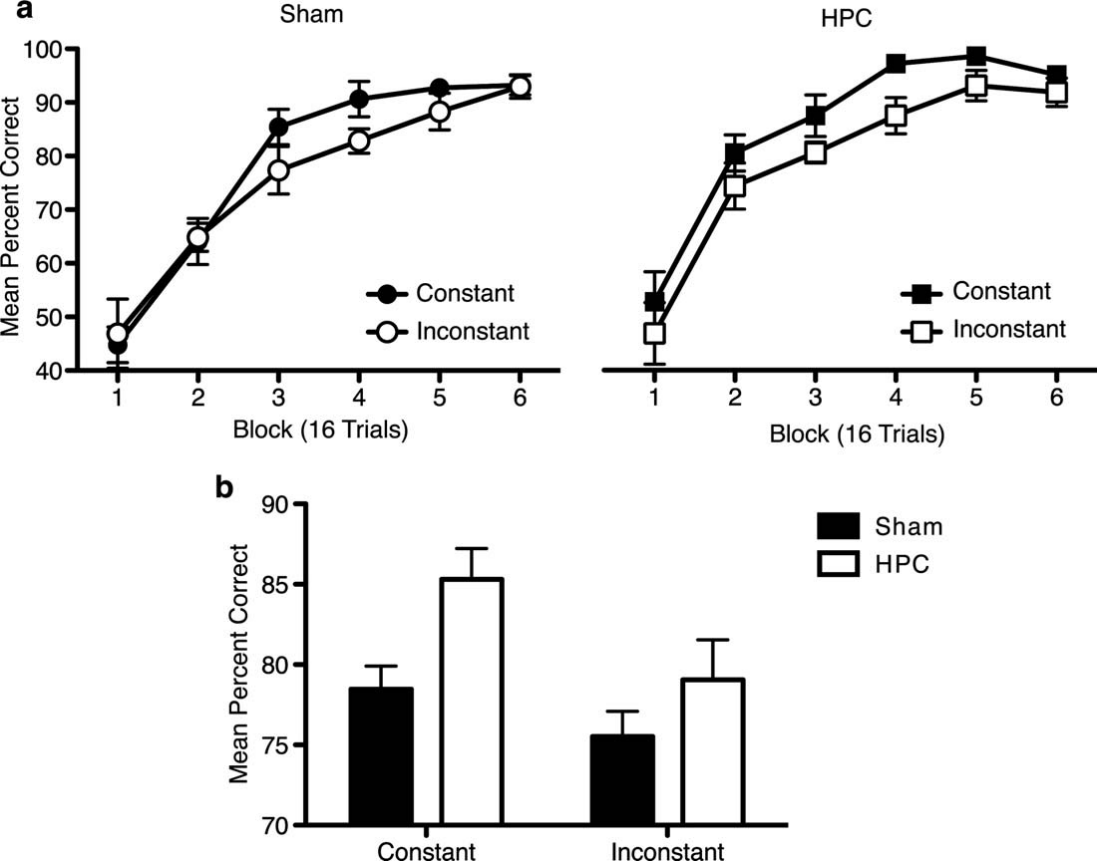
Mean percent correct (±S.E.M.) in the acquisition phase of Experiment 1. (a) Performance is shown in blocks of 16 trials (two sessions). The performance of sham lesioned mice (Sham) in Groups Constant (black circles) and Inconstant (white circles) is shown on the left. The performance of hippocampal lesioned mice (HPC) in Groups Constant (black squares) and Inconstant (white squares) is shown on the right). (b) The overall level of performance over acquisition training for hippocampal (HPC) and sham (Sham) lesioned mice in the Constant and Inconstant conditions. Hippocampal lesioned mice performed at a significantly higher level than sham lesioned mice. Also, Group Constant solved the discrimination at a significantly higher level than Group Inconstant. The effect of lesion did not significantly interact with training condition.

**Figure 4:**
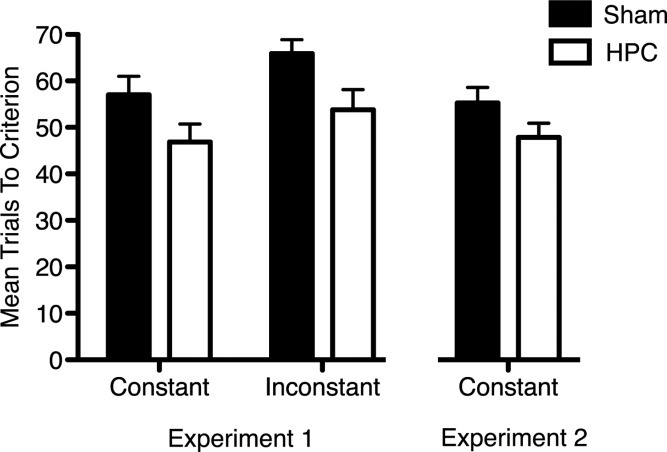
The mean number of trials (±S.E.M.) to reach a criterion of 26 of 32 trials correct across four sessions in the acquisition phases of Experiments 1 and 2. The results of Experiment 1 are shown on the left and the results of Experiment 2 are shown on the right. In Experiment 1 hippocampal lesioned mice (HPC) reached criterion in significantly fewer trials than sham lesioned mice (Sham). Also, mice in Group Constant reached criterion in fewer trials than mice in Group Inconstant. In Experiment 2 mice were trained on the Constant condition. Once again hippocampal lesioned mice reached criterion in fewer trials than sham lesioned mice.

The performance of sham and hippocampal lesioned mice (HPC) in Groups Constant (Sham, *N* = 12; HPC, *N* = 9) and Inconstant (Sham, *N* = 8; HPC = 10) is shown in Figure 3 in blocks of two sessions (i.e., 16 trials). Overall, Group Constant showed greater discrimination learning than Group Inconstant. Also, hippocampal lesioned mice showed greater discrimination in the two conditions compared to sham lesioned mice. These results were confirmed by a 2 (lesion: sham, HPC) by 2 (training condition: constant, inconstant) by 6 (trial block) ANOVA. There was a significant effect of block (*F*(5, 175) = 121.5, *P* < 0.0005), significant effect of lesion (*F*(1,35) = 7.3, *P* < 0.02) and training condition (*F*(1,35) = 5.7, *P* < 0.03). There were no significant interactions between factors (all *P* values > 0.1). The superior performance of hippocampal lesioned mice over control mice was also evident when calculating the number of trials to reach a criterion of 26 correct trials out of 32 (81% correct) across four consecutive sessions (effect of lesion: *F*(1,35) = 7.7, *P* < 0.01; effect of training condition: *F*(1,35) = 3.9, *P* = 0.057; lesion by condition interaction: *F* < 1, Fig. 4).

#### Probe test 1

**Figure 5:**
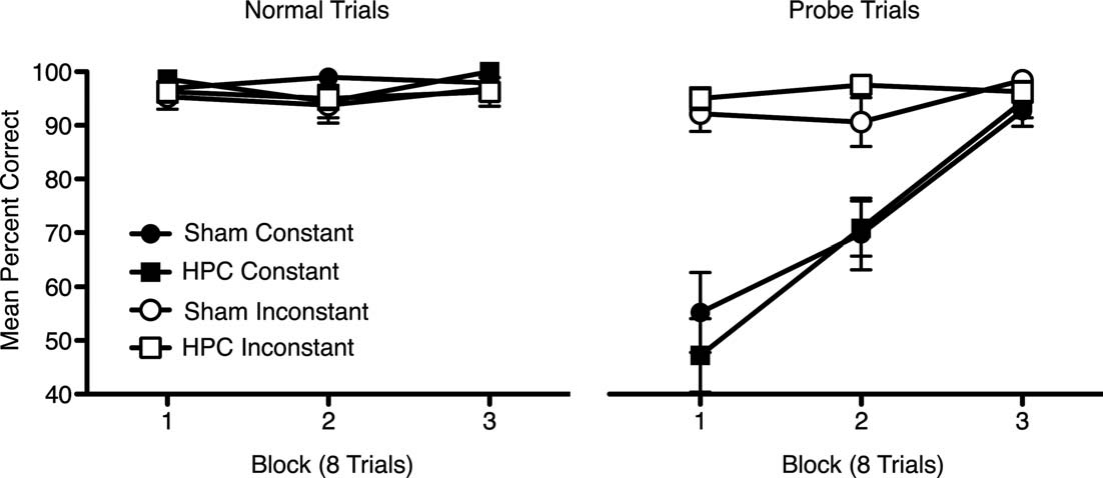
Mean percent correct (±S.E.M.) in probe test 1, Experiment 1. Performance is shown in blocks of eight trials (two sessions) per trial type (normal trials, probe trials). Performance on the normal trials is shown on the left and performance on the probe trials in shown on the right. Both sham and hippocampal lesioned mice in Group Constant (black circles and squares, respectively) performed significantly worse on the probe trials than on the normal trials. Sham and hippocampal lesion mice in Group Inconstant (white circles and squares, respectively) showed a high level of performance on both trials types.

When mice in the Group Constant were switched to the Inconstant condition (see Fig. 1b) performance on the novel, probe trials, in which the stimuli were now presented in the opposite orientation, was initially poor, falling to, or near to chance levels, but improved over further training. Both sham and hippocampal lesioned mice showed a similar sensitivity to the probe trials (see Fig. 5). A 2 (lesion: sham, HPC) by 2 (training condition: constant, inconstant) by 2 (trial type: normal, probe) by 3 (block) ANOVA demonstrated a significant three way interaction between trial type, training condition and block (*F*(2,70) = 17.3, *P* < 0.0005). Separate ANOVAs for each trial type showed that there was no significant interaction between training condition and block for normal trials (*F* < 1), nor any significant effect of block and training condition (both *P* values > 0.1). For probe trials there was a significant training condition by block interaction (*F*(2,70) = 21.4, *P* < 0.0005). Simple main effects analysis showed that there was a significant effect of block for mice trained in the constant condition (*F*(2,34) = 67.0, *P* < 0.0005), but not mice trained in the inconstant condition (*F* < 1). There was also a significant effect of training condition on block 1 (*F*(1,35) = 51.3, *P* < 0.0005) and block 2 (*F*(1,35) = 20.8, *P* < 0.0005), but not on the final block (*P* > 0.15). The effect of lesion was not significant and did not significantly interact with other factors (all *P* values > 0.2)

#### Probe test 2

Performance was of a similar high level on normal trials and the probe trials, with all animals choosing correctly on the vast majority of trials in which the rewarded and nonrewarded stimuli were presented in novel combinations (effect of trial type: *F* < 1). There was no significant effect of training condition (*F* < 1). However, there was again a significant effect of lesion, similar to that which occurred during the acquisition phase, in which hippocampal lesioned mice outperformed sham lesioned mice (Sham = 95% correct ±1.2 S.E.M., HPC = 98.7% correct ±0.6 S.E.M., *F*(1,35) = 7.63, *P* < 0.01). There were no significant interactions between factors (*F* values ≤ 1).

The efficacy of the hippocampal lesions was confirmed by testing on spontaneous alternation. Hippocampal lesioned mice were significantly impaired (Sanderson et al., 2009).

### Experiment 2

#### Acquisition phase

**Figure 6:**
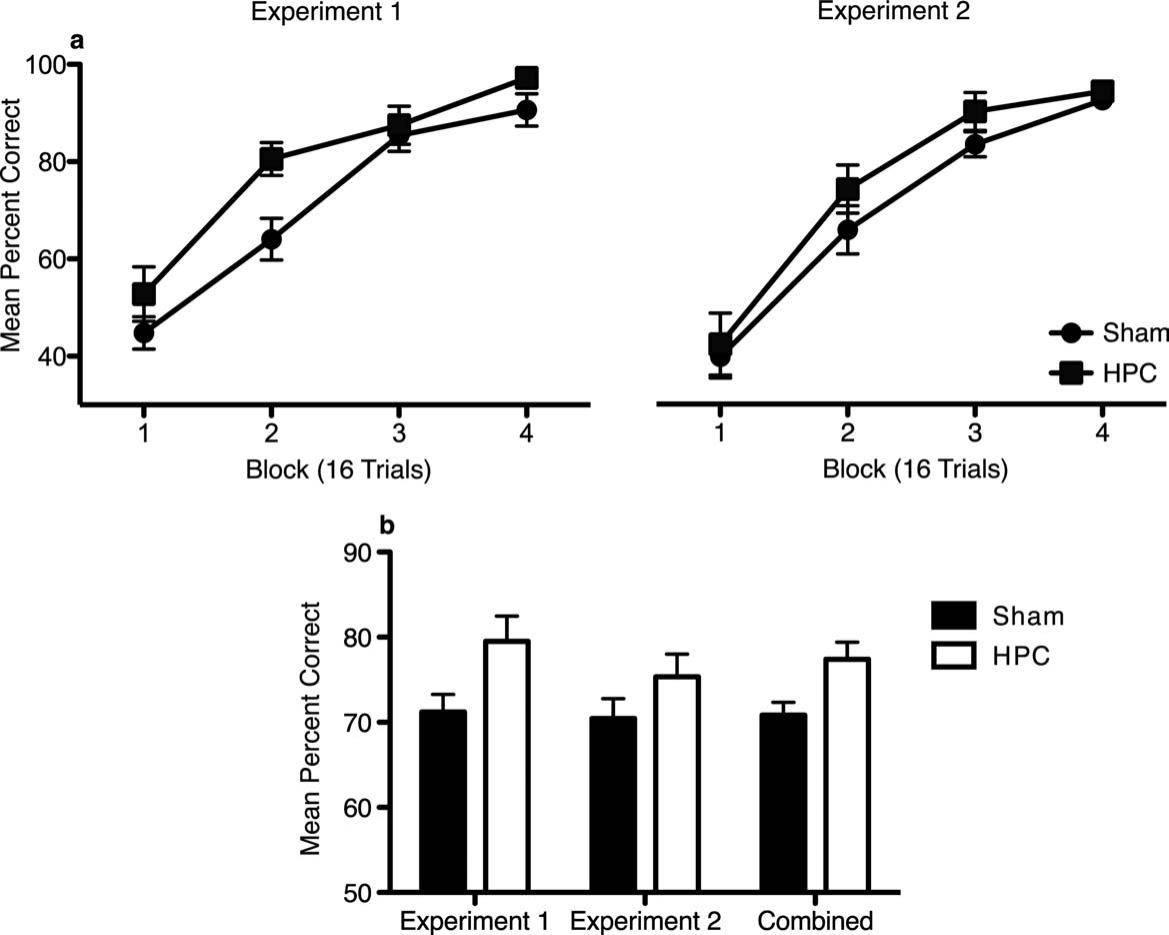
(a) The mean percent correct (±S.E.M.) for the four blocks (16 trials) of acquisition training in Experiment 2 is shown on the right and the equivalent first four blocks of acquisition training for sham and hippocampal lesioned mice (HPC) in Group Constant in Experiment 1 is shown on the left. (b) The overall mean percent correct (±S.E.M.) collapsed across the four blocks of acquisition training in Experiment 2 and the equivalent first four blocks of acquisition training for sham and hippocampal lesioned mice (HPC) in Group Constant in Experiment 1, and the combined performance of sham and hippocampal lesioned mice across both Experiments. Combined analysis of the data from Experiment 1 and 2 showed that hippocampal lesioned mice solved the discrimination to a significantly greater extent than sham lesioned mice. The factor of experiment (i.e., replication) was not significant and did not significantly interaction with the lesion effect.

Sham (*N* = 11) and HPC mice (*N* = 9) acquired the discrimination over the course of training (effect of (16 trial) block: *F*(3,54) = 77.3, *P* < 0.0005) with both lesion groups showing a mean performance greater than 90% correct on the last block of training (Fig. 6a, right panel). The data closely resembled that obtained from Group Constant in Experiment 1 (see Fig. 6a left panel), with hippocampal lesioned mice showing performance that was numerically greater than sham lesioned mice. While there was no significant overall effect of lesion (*F*(1,18) = 1.9, *P* > 0.1) nor interaction of factors (*F* < 1), analysis of the number of trials to reach a criterion of 26 correct trials out of 32 over four consecutive sessions revealed that the hippocampal lesioned mice again reached criterion in fewer trials than the sham lesioned mice (t(18) = 2.07, *P* = 0.05, Fig. 4).

Additional analyses that combined the acquisition training data from Group Constant, Experiment 1 with the acquisition training data in Experiment 2 confirmed that the facilitated performance of hippocampal lesioned mice was reliable across experiments. Thus, an analysis that compared the percent correct across the four blocks of acquisition training in Experiment 2 with the equivalent, first four blocks of acquisition training in Experiment 1 (see Fig. 6a) demonstrated a significant effect of lesion (*F*(1,37) = 7.07, *P* < 0.02), but no significant effect of Experiment (i.e., Experiments 1 and 2; *F* < 1) and no significant lesion by Experiment interaction (*F* < 1; see Fig. 6b). The effect of Experiment did not interact with any other factors (*F* values ≤ 1, *P* values > 0.3). Similarly, an analysis of the trials to criterion demonstrated a significant effect of lesion (*F*(1,37) = 6.62, *P* < 0.02), but no significant effect of Experiment (*F* < 1) or interaction of factors (*F* < 1; see Fig. 4).

#### Probe test

**Figure 7:**
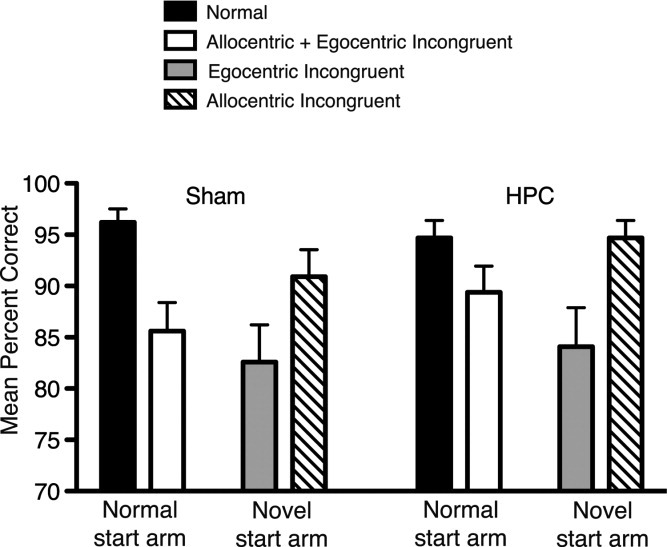
Mean percent correct (±S.E.M.) in probe test 1, Experiment 2. Performance is shown for 12 trials (six sessions) of the different trial types starting from the normal start arm as used in training (normal trials, allocentric + egocentric incongruent trials) and the trial types starting from the novel start arm (egocentric incongruent trials and allocentric incongruent trials) for sham (Sham) and hippocampal lesioned mice (HPC). Mice were significantly worse on egocentric incongruent trials and allocentric + egocentric incongruent trials in comparison to performance on normal trials. Performance was also significantly worse on egocentric incongruent trials compared to allocentric incongruent trials.

Overall, both groups showed worse performance on the egocentric incongruent and allocentric + egocentric trials than on the normal and allocentric incongruent trials (Fig. 7). The data were analysed using a 2 (lesion: sham, HPC) by 4 (trial type: normal, allocentric + egocentric incongruent, egocentric incongruent, allocentric incongruent) by 3 (block) ANOVA. There was a significant effect of trial type (*F*(3,54) = 8.31, *P* < 0.0005) and block, but the interaction between these factors was not significant (*P* > 0.1). The effect of lesion was not significant (*P* > 0.1) and did not significantly interact with trial type (*F* < 1). There was a trend for hippocampal lesioned mice to show better performance on the second block of testing, but the interaction failed to reach significance (*P* = 0.08).

The effect of trial type was analysed using *t*-tests, with the Bonferroni correction for multiple comparisons. It was found that, similar to the results of Experiment 1 (probe test 1), performance on normal trials was significantly greater than on allocentric + egocentric incongruent trials (*P* < 0.005, see Fig. 6). Performance on normal trials was also significantly greater than on egocentric incongruent trials (*P* < 0.005). All other comparisons were not significant (*P* > 0.1, see Fig. 6).

It is possible that the poor performance on the egocentric incongruent trials reflects the fact that mice started from a novel start arm during these probe trials. Therefore, performance may reflect disruption caused by the novel cues associated with that start arm rather than the specific effect of incongruent egocentric information. If this were the case then it would be expected that performance would also be similarly poor on allocentric incongruent trials in which mice also started from a novel start arm and the allocentric information was altered. To test directly whether performance was affected simply by starting mice from a novel start arm an ANOVA was conducted in which trials starting from the original start arm used in training (normal trials, allocentric + egocentric incongruent trials) were compared to trials starting from the novel start arm (allocentric incongruent, egocentric incongruent). The factor of congruency of egocentric information was also included such that trials in which egocentric information was congruent (normal trials, allocentric incongruent trials) were compared to trials in which egocentric information was incongruent (allocentric + egocentric incongruent trials, egocentric incongruent trials). A 2 (start arm: normal, novel) by 2 (egocentric information: congruent, incongruent) ANOVA was used to analyse the factorial design. It was found that there was a significant effect of start arm (F1,18) = 5.79, *P* < 0.03) and a significant effect of egocentric information (*F*(1,18) = 13.83, *P* < 0.003). No interactions were significant (*F* values < 1). This demonstrates that although performance was lower when tested from the novel start arm, the effect of congruency of the egocentric information was independent of the effect of start arm.

A similar ANOVA that tested the congruency of allocentric information instead of egocentric information (allocentric congruent: normal trials, egocentric incongruent trials; allocentric incongruent: allocentric + egocentric incongruent trials, allocentric incongruent trials) as well as the effect of start arm (normal: normal trials, allocentric + egocentric incongruent trials; novel: egocentric incongruent trials, allocentric incongruent trials) found that the effect of start arm significantly interacted with the allocentric information (*F*(1,18) =13.83, *P* < 0.003). Simple main effects analysis of the interaction demonstrated that there was a significant effect of the allocentric information for trials starting from both the original and the novel start arms. For trials starting from the original start arm performance was significantly lower when the allocentric information was incongruent (normal trials vs. allocentric + egocentric incongruent trials, *F*(1,18) = 18.16, *P* < 0.0005). However, for trials starting from the novel start arm performance was significantly greater when the allocentric information was incongruent than when it was congruent (allocentric incongruent trial vs. egocentric incongruent trials, *F*(1,18) = 6.6, *P* < 0.02). The fact that the congruency of allocentric information had opposite effects in the trials starting from the different start arms suggests that mice were not impaired on the allocentric + egocentric incongruent trials due to disruption of the allocentric information, but due to the disruption of the egocentric information. The fact that mice performed worse on egocentric incongruent trials than on allocentric incongruent trials suggests that incongruent egocentric information had a disruptive effect on performance over and above that caused by starting from a novel arm. These results demonstrate that the poor performance on egocentric incongruent trials reflects the specific disruption of egocentric information.

The efficacy of the hippocampal lesions was confirmed by testing on a spatial reference memory task. Hippocampal lesioned mice were significantly impaired (data not shown, manuscript in preparation).

## GENERAL DISCUSSION

The results of Experiments 1 and 2 demonstrate that mice learn the spatial properties of cues despite being incidental to the demands of a simultaneous discrimination task. In Experiments 1 and 2 mice trained in Group Constant initially showed poor transfer of learning when the cues were presented in the opposite spatial arrangement. This suggests that a stimulus that was presented in one spatial location was not treated the same when it was presented in a different location. The probe test in Experiment 2 provided evidence that this effect was primarily due to disruption of egocentric spatial information rather than allocentric information (Fig. 7). Thus, when the allocentric information was altered but the egocentric information remained constant there was not a significant disruption of performance. However, when egocentric information was altered but allocentric information was kept constant there was a reduction in the extent to which the discrimination was solved. Importantly, incidental encoding of irrelevant spatial information impaired acquisition of the discrimination task. In Experiment 1 Group Inconstant showed worse discrimination than Group Constant due to cues being presented in different spatial locations during training.

### Hippocampal Lesions Facilitate Discrimination Learning Independently of Interference Level

Hippocampal lesions significantly enhanced acquisition of the simultaneous discriminations in Experiments 1 and 2. Importantly, in Experiment 1 the significant enhancement caused by hippocampal lesions failed to interact with the training condition (i.e., Constant or Inconstant). This suggests that the hippocampal lesions did not necessarily enhance acquisition by reducing interference from incidental spatial information. However, we cannot rule out the possibility that the failure to find an interaction between lesion and training condition occurred because although the spatial interference was less in Group Constant than in Group Inconstant, the interference may still have been sufficient in Group Constant to result in a beneficial effect of hippocampal lesions. Thus, while spatial information may have interfered with learning in Group Inconstant by causing a generalization decrement between presentations of the same cue in different locations, spatial information may have also resulted in the addition of common features to cues that predict different outcomes (e.g., insert A+ in the left goal is somewhat similar to insert C− when presented in the left goal arm by virtue of being presented in the same goal arm). In Group Constant the addition of common spatial information to cues that predict different outcomes may have been sufficient to result in hippocampal lesions facilitating learning.

This possibility is unlikely given the fact that hippocampal lesioned mice in Group Constant showed a reduction in performance to near chance levels on probe trials in which the spatial arrangement of cues was reversed, that was similar to that seen in control mice (Experiment 1 and 2). If hippocampal lesions had reduced the interference from spatial cues in Group Constant then it would have been expected that hippocampal lesioned mice would have been less affected by the change in spatial arrangement in probe test 1 of Experiment 1, and would have shown superior performance on these probe trials. The lack of effect of hippocampal lesions on these probe trials is striking given that hippocampal lesions significantly enhanced the initial learning of the task. Therefore, it is unlikely that the absence of a hippocampal lesion effect reflects insensitivity of the measure or lack of power.

Both hippocampal lesioned mice and control mice were insensitive to the novel pairings of rewarded and nonrewarded stimuli (Experiment 1, probe test 2). This suggests that rewarded and nonrewarded cues were not treated as a configuration, and that they were encoded independently from one another. The lack of effect of hippocampal lesions on solving the discrimination with novel combinations of rewarded and nonrewarded stimuli is consistent with other findings suggesting that approach and response behavior is guided by simple associations formed between stimuli and an outcome (Deacon and Rawlins, 1996; Driscoll et al., 2004).

### Facilitated Learning in Hippocampal Lesioned Mice Does not Necessarily Reflect Qualitatively Different Learning

The results of the probe tests in Experiments 1 and 2 fail to provide a clear account of the facilitated performance in hippocampal lesioned mice. The normal encoding of egocentric spatial information in hippocampal lesioned mice demonstrates that it is unlikely that hippocampal lesions facilitated performance by reducing interference from incidental spatial information. Specifically these results fail to support the theory of hippocampal function proposed by Gluck and Myers (1993). This theory suggests that the hippocampus modulates the representation of stimuli that is encoded due to its bias for “predictive differentiation” in which the discriminability of stimuli that predict different outcomes is enhanced, and “redundancy compression” in which the differences between stimuli that predict the same outcome are reduced. These two biases result in stimulus representations that change with experience, whereas the stimulus representation is fixed in the absence of the hippocampus. The fact that stimulus representations are modified with experience by the hippocampus results in a certain amount of generalization decrement across trials in controls, that reduces acquisition of simple discriminations in comparison to learning in the absence of the hippocampus.

Gluck and Myer's account assumes that hippocampal lesions facilitate discrimination learning because they reduce the information that is encoded. Thus, the nature of the stimulus representation will be different between hippocampal lesioned animals and controls. The results of Experiments 1 and 2 fail to provide evidence for this account. Although it is possible that the facilitation is due to hippocampal lesions reducing the rate at which the egocentric spatial information is encoded, the lack of a lesion by block interaction in the acquisition phases of Experiments 1 and 2 suggests that this is not the case.

### The Role of Egocentric Responses Versus Egocentric Representations in Discrimination Learning

The finding that hippocampal lesions spare acquisition of egocentric spatial information is consistent with other findings in rodents (Oliveira et al., 1997; DeCoteau and Kesner, 2000; Ramos and Vaquero, 2000; Rogers and Kesner, 2006) (see also Nadel and Hardt, 2004; Burgess, 2008). It is typically assumed that disruption of hippocampal function spares learning of egocentric responses such as “turn left” and “turn right.” Indeed rats with hippocampal lesions (DeCoteau and Kesner, 2000; Ramos and Vaquero, 2000) or inactivation of the hippocampus (Packard and McGaugh, 1996) show a greater reliance on egocentric responses than control rats. In the present study, however, it is not possible to tell whether the egocentric spatial learning reflected the use of egocentric responses or learning of the relative egocentric position of the cue to the mouse's body. It may be more likely that the latter is the case, because if mice had learnt egocentric responses, (e.g., A+ on the left elicits a turn left response), then it might be expected that Group Constant would perform below chance when the cues were presented in the opposite spatial arrangement (Experiment 1, probe test 1; Experiment 2, probe test 1, allocentric + egocentric incongruent trials). Thus, when cues are presented in the opposite spatial arrangement it would be expected that a cue would still elicit the same egocentric response, although to a lesser extent due to generalization decrement, which would result in mice entering the wrong goal arm (e.g., cue A+ on the right elicits a go left response). This was found not to be the case. Although performance started at chance levels in probe test 1 in Experiment 1, mice rapidly learnt to approach the correct insert. Furthermore, in Experiment 2, performance was above chance at the start of testing on these probe trials (Block 1: Sham – 82% correct, HPC – 83% correct, allocentric + egocentric incongruent trials, Experiment 2). The superior performance on these probe trials in Experiment 2 compared to Experiment 1 may reflect that mice were trained on the initial task for longer in Experiment 1, or it may reflect a beneficial effect of the additional probe trials (e.g., egocentric incongruent and allocentric incongruent) in Experiment 2. However, the fact that performance in the two experiments was not below chance is consistent with an account in which mice learnt to simply approach or to avoid cues and generalization of these approach and avoidance responses was dependent on the relative position of the cue to the mouse's body.

### The Hippocampus and Incidental Learning

The spared incidental encoding of egocentric spatial information in hippocampal lesioned mice contradicts the predictions of a theory of hippocampal function proposed by O'Reilly and Rudy (2001). O'Reilly and Rudy suggested that the hippocampus is necessary for rapid learning of conjunctions between cues when they are incidental to the demands of the task. In the present experiments hippocampal lesions failed to impair learning of the conjunctions between the insert cues and their relative egocentric position. The egocentric spatial information was incidental: it was not necessary for acquisition of the task and, furthermore, was at a cost to performance (Experiment 1, Group Inconstant). It is possible that hippocampal lesioned mice were facilitated because they failed to encode the incidental information as rapidly as controls. However, this post hoc account of performance would not necessarily be predicted by O'Reilly and Rudy's model. At the very least the present results are in contrast to a number of examples of impaired incidental learning in hippocampal lesioned rodents (Save et al., 1992; Honey and Good, 1993; Good and Bannerman, 1997; Mumby et al., 2002; Good et al., 1998, 2007; Langston and Wood, 2010), suggesting that the hippocampus is not necessary for all forms of incidental learning (Honey and Good, 2000; Coutureau et al., 2002; Ward-Robinson et al., 2001, 2005).

### Do Hippocampal Lesions Facilitate Nonspatial Discrimination Learning by Impairing the Inappropriate use of Spatial Strategies?

It is possible that sham lesioned mice showed worse performance than hippocampal lesioned mice because they initially persisted with an incorrect spatial strategy (e.g., spatial alternation), dependent on allocentric cues, before then adopting the correct cue-based solution to the task (Meunier et al., 1996; Saksida et al., 2007). This account is in keeping with the idea that lesions of the hippocampus reveal the existence of competing learning mechanisms (White and McDonald, 1993; Ito et al., 2005, 2006). This account predicts that hippocampal lesions would facilitate performance regardless of the training condition and that the sham and lesioned mice would eventually learn the same information. The results of Experiments 1 and 2 confirm these predictions. In Experiment 1 hippocampal lesions enhanced performance independent of the training condition, and the probe trials in Experiments 1 and 2 confirm that both the sham and hippocampal lesioned mice learnt the same qualitative information. This account, however, would not necessarily anticipate that either of the sham and hippocampal lesioned groups would be affected by the probe trials in which the spatial information was altered. It would assume that once the task has been learnt that spatial cues are treated as irrelevant and animals would adopt a purely cue-based strategy. However, in Experiments 1 and 2 mice integrated spatial information and cue information. This demonstrates that spatial and nonspatial learning systems do not function entirely independently of one another, and for the case of egocentric spatial information the integration of information does not occur in the hippocampus.

While the design of Experiments 1 and 2 does not provide a direct test of whether control mice persist with a spatial strategy before adopting a cue-based strategy there is evidence that allocentric spatial cues can overshadow the use of landmarks in navigation tasks in normal animals (Diez-Chamizo et al., 1985; March et al., 1992). Thus, it is possible that allocentric spatial cues may retard the use of cue-based strategies more generally. An experiment by Ramos (2001) provides further support for this argument. Sham and hippocampal lesioned rats were trained on a spatial task in which both extramaze and intramaze cues indicated the location of reward. In a probe test in which only the intramaze cues were available to signal the location of reward it was found that hippocampal lesioned rats performed significantly better than control rats. Thus, hippocampal lesions facilitated performance presumably because control rats relied primarily on a strategy using the extramaze cues.

The idea that hippocampal lesions can enhance learning of nonspatial tasks due to control animals persisting with incorrect spatial strategies provides a simple explanation of the current results. However, it fails to explain why hippocampal lesions can also impair learning on some nonspatial tasks. For example, it has been found that damage to the hippocampal system impairs recency-dependent memory (Fortin et al., 2002; Kesner et al., 2002; Charles et al., 2004; Marshall et al., 2004), the expression of memory for associations between auditory and visual stimuli (Honey et al., 1998; Honey and Good, 2000), timing (Meck et al., 1984; Sinden et al., 1986; Reisel et al., 2005) and cost-benefit decision making (Mariano et al., 2009). In all these examples there is either no obvious spatial component or spatial information was made irrelevant for the solution of the task. Despite this, impairments following hippocampal system damage were found. This suggests, in light of the present experiments, that facilitated learning caused by hippocampal lesions on nonspatial tasks may not necessarily reflect faster learning due to a lack of spatial learning. Thus, it is not clear why a lack of spatial learning might lead to facilitation on some nonspatial tasks, but actually lead to deficits on other nonspatial tasks. Collectively, these results suggest that the hippocampal system is necessary for more than just spatial learning.

In conclusion, hippocampal lesions can facilitate learning, but this does not necessarily occur due to impairing encoding of the incidental properties of the stimuli. Hippocampal lesions spare the acquisition of egocentric spatial information and do so even when the information is incidental to the demands of the learning tasks and is detrimental to performance.
